# Experiments on the Effects of Nicotine

**Published:** 1851-10

**Authors:** 


*Experiments on the Effects of Nicotine.*—This alkaloid, which has acquired some renown from the crime and execution of Count Bocarmé, in Belgium, was administered to various animals by M. Vleminckx, of Brussels. The following results were obtained:—

1. One drop killed a sparrow in twenty five seconds. 2. Half a drop destroyed another sparrow in forty seconds. 3. Two drops killed a cock of large size in forty seconds; the same effect being produced in a minute, by the same dose, upon a rabbit. 4. A small dog died in two minutes, thirty seconds, after taking two drops of nicotine, dissolved in ten of sulphuric ether. 5. Four drops, in the same menstruum, killed a cat in three minutes. 6. One drop was placed on the conjunctiva of a middle-sized dog; vertigo, weakness, and trembling ensued, and the cornea became hazy and rough. After six minutes he recovered. Two drops were then put upon the tongue of the same dog: he soon fell, after tottering about, and eventually recovered. Another and larger dog had ten drops: the symptoms were of the convulsive character. Vinegar was poured down his throat, and he regained his powers soon afterwards, though he threw up the vinegar.

The inspections proved that the most constant pathological changes were, congestion of the vessels of the pia mater, and especially a very intense congestion of the lungs.—*London Lancet.*

